# The War on Impure Food

**Published:** 1911-03

**Authors:** 


### The War on Impure Food

DURING the past month Health Commissioner Fronczak, ably aided by Dr. W. H. Heath, the health department's chief inspector of foods, has been waging a relentless warfare upon the dealers in canned, frozen and otherwise unfit eggs. During the investigation which has been conducted with a vigor commendable from every standpoint it has been definitely shown that there have been frequent instances in which eggs, which were rather more advanced than merely spoiled, have been sold to bake shops and candy manufacturers for use in the production of cakes and ice creams.

Numerous technicalities were injected into the hearings and some of the terms explanatory of the physical condition of eggs at various stages of their careers carried elements of humor which one would scarcely expect in a commonplace commercial life. There are "spots," and "rots" and "leaks" and "cracks" all of which in themselves mean nothing to the casual observer at a distance but which actually cover not only a multitude of days and months of past usefulness but even serve to disguise everything of their actual condition save the smell and this is said in a measure to have been dulled by the freezing process.

There were persistent rumors of "financial diplomacy" which stripped of its fanciful trimming means no more nor less than commonplace bribery of health department officials. Of course there were denials and the vigor with which the rumors were traced down by the department is the best indication of the official cleanliness of the inspectors. The investigation is not completed at this writing but it has already borne excellent results. The rotten egg market has taken a decided slump and there is every indication that a better grade of ingredients is being used in the manufacture of the foods which are sold in Buffalo.

Dr. Fronczak is doing splendid work and is ably following up the crusades which were inaugurated by the late Ernest Wende, and it is largely due to Dr. Heath's persistent endeavor that so much progress has been made in the correction of glaring evils and the suppression of much of crookedness on the part of food stuff dealers.

Uncompromising as is the fight which Dr. Fronczak is waging against impure and dangerous adulterated food products he cannot hope to succeed without the honest coöperation of the newspapers and the city administration. He cannot hope for the result which his efforts deserve with the insufficient staff at his command and the board of aldermen should give him able assistants sufficient in number to guard the health of the city.

